# A Lightweight Object Detection Network for Real-Time Detection of Driver Handheld Call on Embedded Devices

**DOI:** 10.1155/2020/6616584

**Published:** 2020-12-15

**Authors:** Zuopeng Zhao, Zhongxin Zhang, Xinzheng Xu, Yi Xu, Hualin Yan, Lan Zhang

**Affiliations:** ^1^School of Computer Science and Technology & Mine Digitization Engineering Research Center of the Ministry of Education of the People's Republic of China, China University of Mining and Technology, Xuzhou 221116, China; ^2^School of Computer Science and Technology, China University of Mining and Technology, Xuzhou 221116, China

## Abstract

It is necessary to improve the performance of the object detection algorithm in resource-constrained embedded devices by lightweight improvement. In order to further improve the recognition accuracy of the algorithm for small target objects, this paper integrates 5 × 5 deep detachable convolution kernel on the basis of MobileNetV2-SSDLite model, extracts features of two special convolutional layers in addition to detecting the target, and designs a new lightweight object detection network—Lightweight Microscopic Detection Network (LMS-DN). The network can be implemented on embedded devices such as NVIDIA Jetson TX2. The experimental results show that LMS-DN only needs fewer parameters and calculation costs to obtain higher identification accuracy and stronger anti-interference than other popular object detection models.

## 1. Introduction

Traffic congestion is a huge problem [1] facing the world in the process of rapid urbanization. In order to avoid traffic accidents, algorithms and embedded environment applications of drivers' dangerous behavior detection [2], such as handheld receiving and calling, have been studied extensively [3–7]. Most of the research on object detection networks aims at improving accuracy [8] but ignores the problems of model, calculation amount, and the number of parameters. In 2014, Girshick et al. proposed a region-based convolutional neural network (R-CNN) [9], which uses a region-based recognition method to detect objects. In 2015, two improved versions of R-CNN—Fast R-CNN [10] and Faster R-CNN [11]—were proposed to realize end-to-end detection of targets. Both models are two-stage algorithms with higher accuracy than traditional algorithms, but the detection speed is slow and cannot meet the real-time requirements. In 2016, Redmon et al. proposed YOLO [12] and YOLO9000 [13] and then proposed YOLOv3 [14]; the latter greatly improved the detection effects of small objects when compared to YOLO9000. The YOLO series algorithm greatly improves the detection speed because it combines the two-stage task of sorting and identifying candidate box in Faster R-CNN. However, the detection speed is relatively slow due to the large number of parameters of YOLOv3. The network should be both accurate and fast, in order to realize the operation of the object detection network on mobile devices. Liu et al. then proposed an SSD [15] algorithm to realize the regression detection of the whole image that improved the speed but greatly reduced the accuracy of the detection of small targets. YOLO, SSD, and the network derived from them, as the representatives of the phase network, realize end-to-end training. They only use a convolutional neural network to directly predict the categories and positions of different targets and improve the detection speed [16] by sacrificing accuracy.

Tiny-Yolo, introduced in 2017, is widely used due to its high speed and low memory consumption, but for devices that do not have GPU, it is still difficult to use Tiny-Yolo for real-time applications. In the same year, Andrew G. Howard et al. proposed MobileNet [17] for mobile and embedded vision applications. In 2018, the network MobileNet-SSD, derived from VGG-SSD, was proposed to dramatically reduce the parameters while greatly increasing the detection speed, but the risk of missing and false detection of small objects was extremely high in this. Several efficient mobile neural networks for common object detection have been proposed in the year recently, such as SqueezeNet [18], ShuffleNet [19], and so on. In order to further enhance the accuracy of small object detection and study the possibility of the application of lightweight object detection network in embedded devices, a new lightweight object detection network is proposed in this paper, which especially improves the detection ability of small target objects.

The rest of this paper is organized as follows.  Section 2 mainly introduces the related research on the application of small target detection and deep learning network on embedded devices.  Section 3 introduces the overall structure of MobileNetV2 and SSD.  Section 4 presents the structure of the new object detection network Lightweight Microscopic Detection Network (LMS-DN) and explains the innovation of the network.  Section 5 provides and compares the experimental results with previous object detection networks on the KITTI, VOC, and Safe_Imgs datasets. The experimental results demonstrate the efficiency improvement of LMS-DN implemented on NVIDIA Jetson TX2. Finally, we draw conclusions on this work in Section 6.

The main contributions of this study are as follows:

1. Based on MobileNetV2-SSDLite, a new object detection network LMS-DN is proposed. Among them, the basic network MobileNetV2 is improved, and the network MobileNet-I is proposed. In order to better detect small objects, the other two layers of object features are selectively extracted for detection.
2. This study does not require complex preprocessing of driver status images and achieves considerable results without user intervention.
3. LMS-DN can be implemented in real time on NVIDIA Jetson TX2 embedded devices and can be used for practical testing of different lighting and obstacle occlusion conditions.

## 2. Related Work

Driver's handheld call detection can be regarded as the detection of small target mobile phone, which has become a challenging problem in the field of target detection. If an object is only a small part of an image (less than 0.1% of the image area), it is considered a small target. By analyzing the previous target detection networks (such as R-CNN, R-FCN [20], etc.), it can be said that a large number of convolution layers are used in the network structure to extract image features [21] dynamically. At the same time, the pool layer is designed to gradually reduce the size of the feature map that further reduces the calculation cost and prevents the model from overfitting. In this process, the resolution of the image is obviously reduced, and the information loss for small objects is very high. In view of the above problems, many researchers are focusing on the study of small target detection. Chen et al. proposed TSSD in 2017, which reused the pyramidal feature hierarchy calculated by convolution networks and used each layer of feature maps for detection, but it requires high computational capacity and memory of the computers. Krishna and Jawahar add deconvolution layers to the RPN to restore low-resolution images to high resolution, thus improving the detection effect for small targets. Lin et al. proposed FPN [22], which can be predicted on different feature maps by merging high-level semantic information and low-level location information. This method is very important in small target detection. In the same year, Guimei et al. proposed the feature-fused SSD [23] based on SSD, which fused different levels of features into context information, which is helpful in detecting small objects. IoU threshold is very important for sample selection. If the IoU threshold is too high, the quality of a positive sample will be very high, but the quantity will be very small, and the imbalance in the sample proportion will have a large influence on the final outcome. If the IoU threshold is low, the number of samples will increase, but the quality of samples will decrease. How to balance this relationship, that is, how to select a good IoU, is very important for the detection results. The cascade R-CNN, proposed by Cai et al. in 2018, continuously raised the threshold of IoU through a multistage structure, while ensuring the number and quality of samples, and finally trained a high-quality detector. Singh et al. proposed SNIP, which pretrained the image and then fine-tuned it using the original sampled image, and used the fine-tuned model to predict the original sampled image, thus making the input distribution as close to the model's pretrained distribution as possible. Zhishuai Zhang proposed DES on the basis of SSD, added semantic segmentation branch and global activation module, and carried out high-level target detection features by learning the semantic relationship between feature channels and target categories.

Artificial intelligence and machine learning need a powerful computing platform for its implementation. The computing module is mostly placed at back end. An embedded system is developed for special equipment; one of its characteristics is high cost, so hardware resources are limited. In the face of some special scenes such as automobile driving, the real-time requirement of embedded equipment is high. NVIDIA launched a high-performance Jetson TX2 embedded board to speed up the layout of deep learning on terminal devices. The chip will be used in urban security, smart drones, ocean observations, intelligent agriculture, and industrial and commercial robots. Jetson TX2 really solves the real-time positioning and map building (Simultaneous Localization and Mapping, SLAM) computing requirements. There are several case studies about CNN-based object detection algorithms for embedded platforms. Mao et al. implemented the detection algorithm Fast R-CNN on Jetson TK1. They applied SVD to the FC layer, and by adopting a more effective approach to regional recommendations, simplified the Fast R-CNN model. Both the CPU and GPU were employed to achieve the parallel computing of region proposal and CNN classification. Wang et al. customized the design parameters of the object detection framework, to maximize the algorithm energy efficiency on the Jetson TX1 platform. They achieved a trade-off between CNN accuracy and efficiency by adjusting the input size and hyperparameters of the CNN model. Yu et al. summarized the optimization technology of energy-saving target detection based on CNN on different hardware platforms. They applied SVD decomposition and Winograd's algorithms to reduce CNN computational complexity. The aforementioned works mainly focused on the design of the algorithm to reduce the computational complexity of the CNN model, which is often at the expense of accuracy loss. This paper proposes a lightweight model LMS-DN that needs only a few parameters. The calculation costs to obtain higher identification accuracy are lower than other popular object detection models. LMS-DN can capture the location information of small objects to improve the accuracy of mobile phone detection while running on NVIDIA Jetson TX2 embedded devices in real time.

## 3. Methodology

### 3.1. MobileNetV2 Lightweight Network

**Figure 1:**
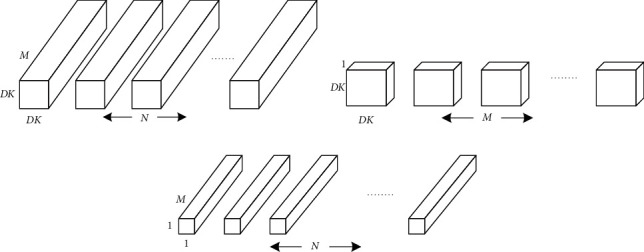
Depth separable convolution. (a) Standard convolution filters. (b) Depthwise convolution filters. (c) 1 × 1 convolution filters called pointwise convolution in the text of depthwise separable convolution.

MobileNetV2 is an improvement upon MobileNetV1. MobileNetV2 can reduce the number of parameters and improve the operational speed more effectively, with the use of depth separable convolution and the incorporation of residual connection, providing an effective model for mobile and embedded visual applications. Depth separable convolution is a decomposable convolution operation that can be divided into two smaller operations: Depthwise Convolution and Pointwise Convolution. The first step uses single-channel convolution to check the convolution of each channel of the input data. In the second step, N Point convolution with the same depth as the input data is used to check the results separated in the previous step and combine them to generate new results, as shown in Figure 1.

**Figure 2:**
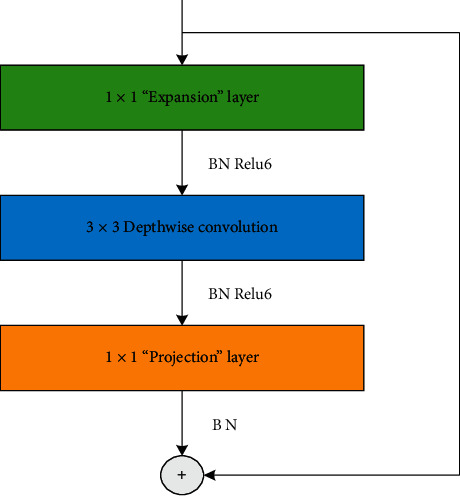
Inverted residual.

Table 1 shows the main structure of MobileNetV2, where *t* represents the “expansion” multiple, C represents the number of output channels, N represents the number of repeats, and S represents the stride length. The core of MobileNetV2 is the bottleneck residual block (inverted residuals), as shown in Figure 2, which forms the “expansion” to “convolutional layer extracts features” to “compression” structure. Expansion layer uses 1 × 1 convolution kernel in order to map the low-dimensional space to the high-dimensional space. Among them, one super parameter is the dimension expansion multiple, which defaults to 6. After the depth separable convolution layer, it is projection layer, and 1 × 1 convolution kernel is also used in order to remap the high-dimensional features to the low-dimensional space. The function of the inverted residual module is to transform the input data into high dimensions and then extract features through deep decomposition and convolution.

### 3.2. Single Shot MultiBox Detector

**Figure 3:**
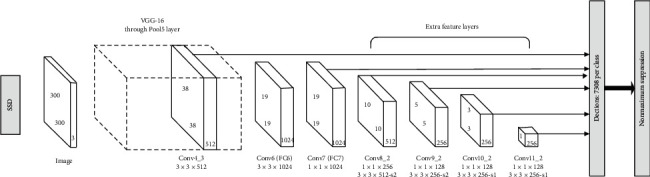
Structure of SSD.

Single Shot MultiBox Detector (SSD) is an object detection network [6] that can directly predict the target category and position. In the previous model, the standard architecture network used for image classification was called the basic network, and then some additional layers were added, and the feature maps output by six different convolutional layers were fused to achieve comprehensive detection.  Figure 3 shows the SSD. VGG16 [4] forms the basic network of the algorithm. The two full connection layers are changed into the convolutional layer, and another four convolutional layers are added to construct the network structure. The six convolutional layers involved in feature map fusion will produce a certain number of borders, which are together called the default box. The calculation formula of default box size is as follows:

(1) $$\begin{array}{c} S_{k}=S_{\min}+\frac{S_{\max}-S_{\min}}{m-1}\left(k-1\right),\;k\in \left[1,m\right], \end{array}$$

where *S*_min_ is 0.2, *S*_max_ is 0.95, through aspect ratio *A* to adjust the default box. In the calculation of loss function, SSD uses the sum of two terms, including positioning loss function and regression loss function. Total loss function:

(2) $$\begin{array}{c} L\left(x,c,l,g\right)=\frac{1}{N}\left(L_{\text{conf}}\left(x,c\right)\right)+\alpha L_{\text{loc}}\left(x,l,g\right). \end{array}$$

*N* is the number of matched default boxes. *x* represents whether the matched boxes belong to category *p*, and the value is {0, 1}. *g* is the ground truth box; *c* refers to the confidence of the checked target belonging to category *P*.

## 4. Proposed LMS-DN

**Figure 4:**
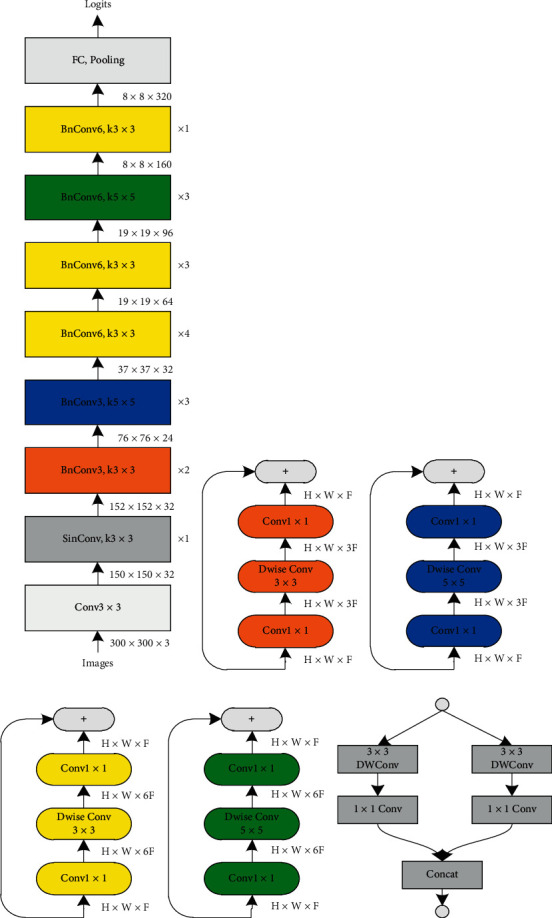
Structure of MobileNet-I. (a) MobileNet model. (b), (c), (d), (e), and (f) Corresponding layers structure for MobileNet-I.

LMS-DN is proposed in order to meet the requirements of real-time detection [24] and to further enhance the extraction of features of small objects. The first part of the two-part LMS-DN is the improved basic classification network, MobileNet-I (the network structure is shown in Figure 4), and the second part is the improved SSDLite network. MobileNet-I borrows from inception [25] structure expansion features to extract sensing field, integrates 5 × 5 depth separable convolution, and adjusts the overall structure of MobileNetV2. The MobileNet-I model contains 5 × 5 depth convolution compared to the previous studies that typically used only a 3 × 3 kernel. In fact, a 5 × 5 kernel does save resources for deeply separable convolutions rather than two 3 × 3 kernels. Formally, given the input shape (*H*, *W*, *M*) and output shape (*H*, *W*, *N*), let C_5×5_ and C_3×3_, respectively, represent the calculation cost of depth separable convolution of 5 × 5 and 3 × 3:

(3) $$\begin{array}{c} C_{5\times 5}=H*W*M*\left(25+N\right),\\ C_{3\times 3}=H*W*M*.7\left(9+N\right),\\ C_{5\times 5}<2*C_{3\times 3},\;\left(if\;N>7\right). \end{array}$$

**Figure 5:**
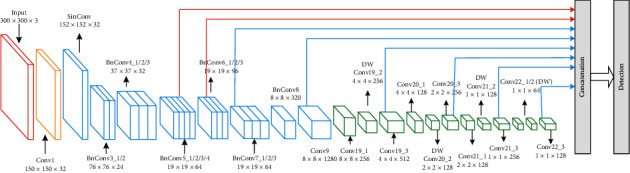
Proposed LMS-DN structure.

For the same effective receptive field, when the input depth is *N* >7, a convolution kernel with 5 × 5 has less computation than two cores with 3 × 3. Remove the last pooling layer of MobileNet-I and add an auxiliary convolutional layer to connect the base network and SSDLite networks. SSDLite replaces some standard convolution in the SSD prediction layer with Deep separable Convolution. According to the feature extraction mechanism of small target and the characteristics of different convolutional layers, LMS-DN extracted two additional features of special convolutional layers to detect the target, and that is very effective for detecting small target objects such as mobile phones. Thus, Figure 5 shows the overall structure of LMS-DN that is formed.

As can be seen in Figure 5, in addition to the six feature maps extracted previously, LMS-DN also selects feature maps of the “expansion” layer in two convolution units, BnConv5_4 and BnConv6_2 for convolution, permutation, and flattening, and then merge the output for detection through the splicing layer. The reasons for the additional extraction of two feature maps of convolutional layers are as follows:

1. From the above definition, when the input image resolution is 300 × 300, the resolution of small objects should not be greater than 30 × 30. In the deep learning model, the deeper the network layer is, the stronger is the ability to extract image features. In order that the features of small objects could be optimally learned without losing information about them, we decide to extract the feature map of the “expansion” layer of the convolution unit BnConv5_4 with an image no greater than 30 × 30.
2. The resolution of the convolution unit BnConv6_2 is 19 × 19. As the transition layer of convolution units BnConv5_4 and BnConv7_1, BnConv6_2 has information that is more global, and with better feature extraction capability, it is more conducive to detecting small targets in images.

## 5. Experimental Results and Analysis

### 5.1. Dataset and Experimental Settings

In this paper, the experimental platform for Intel Core I5 7200U processor, NVIDIA GTX 1080 8G memory, software environment for Ubuntu16.04, Caffe's [26] deep learning framework, using VOC2007 and VOC2012 datasets [27], KITTI dataset [28] to training models and test the performance of LMS-DN, and other popular object detection networks have been described. Finally, the Safe_Imgs collected in the driver video monitoring platform have been used to detect the handheld call of the driver through LMS-DN.

VOC2007 and VOC2012 datasets are divided into 20 target object categories and 21 background categories. In this paper, both VOC2007 and VOC2012 are taken as training datasets while VOC2007 test sets alone are used for testing.

KITTI datasets contain real image data that have been collected from urban, rural, and highway scenes and are annotated in eight categories. In this paper, in KITTI dataset, person sitting is merged into a pedestrian. The dataset consists of seven categories: car, van, truck, tram, pedestrian, background, and so on. Of the 7,400 tagged images, 6,660 were used as training sets and 740 as test sets.

**Figure 6:**
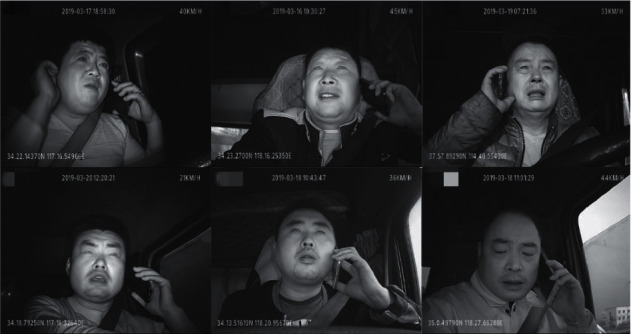
Safe_Imgs dataset.

All images of the Safe_Imgs dataset are from the driver video monitoring platform [29], as shown in Figure 6. The safe driving and talking video of driving of 30 drivers were selected in the data, out of which the driving videos of 15 drivers were selected as the verification set. Using OpenCV to capture the collected videos at 10 frames per second with a resolution of 300 × 300, 5,500 images of the training dataset were obtained, and 550 images of the dataset were verified. LabelImg [30] was used to generate an XML file corresponding to the sample after annotation.

### 5.2. Performance Evaluation Indicators

There are many evaluation criteria for the object detection algorithm. According to the emphasis of different standards, this paper uses detection accuracy, detection efficiency, and model size to evaluate the object detection model. The accuracy, precision rate, recall rate, mean average precision (mAP) were used to evaluate the performance of the target detection model in all categories on the dataset. The calculation methods of these indexes are as follows:

(4) $$\begin{array}{c} \text{accuracy}=\left(1-\frac{a}{m}\right)\times 100\%,\\ \text{precision}=\frac{\text{TP}}{\text{TP}+\text{FP}}\times 100\%,\\ \text{recall}=\frac{\text{TP}}{\text{TP}+\text{FN}}\times 100\%. \end{array}$$

where *a* is the number of misclassified samples, *m* is the total number of samples, TP (true positive) refers to a positive sample that is predicted to be positive by the model, FP (false positive) refers to a negative sample that is predicted to be positive, and FN (false negative) refers to a positive sample that is predicted to be negative. Frames per second (FPS) was used to evaluate the detection efficiency; and MB (MByte) was used to evaluate the size of the model. By weighing these performance indexes, the algorithm which is more suitable for embedded transplantation is discussed.

### 5.3. Results and Analysis

**Figure 7:**
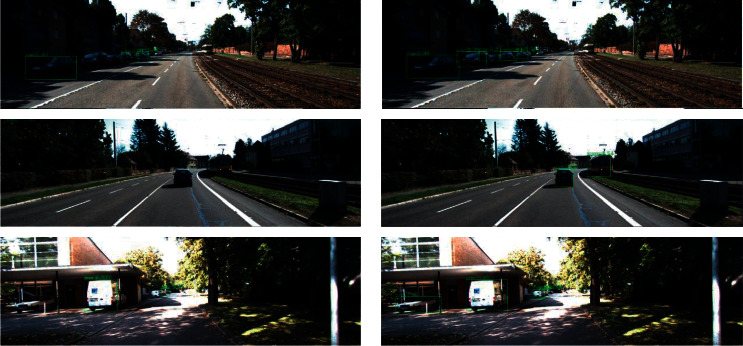
Effect comparisons on Safe_Imgs dataset. (a) LMS-DN. (b) MobileNetV2-SSDLite.

**Figure 8:**
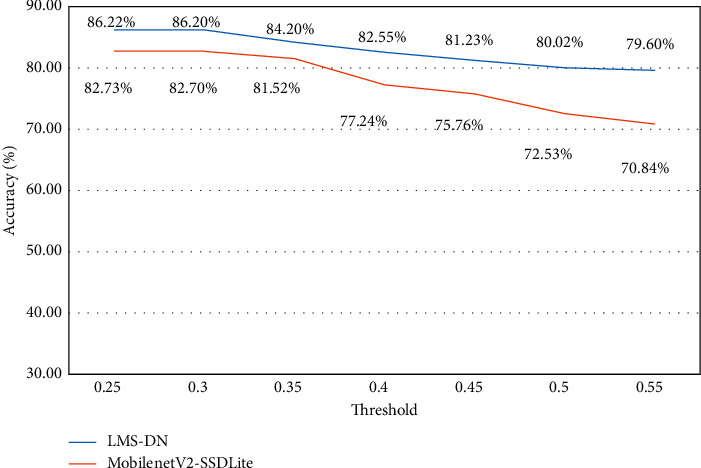
Accuracy of the networks in different thresholds.

**Figure 9:**
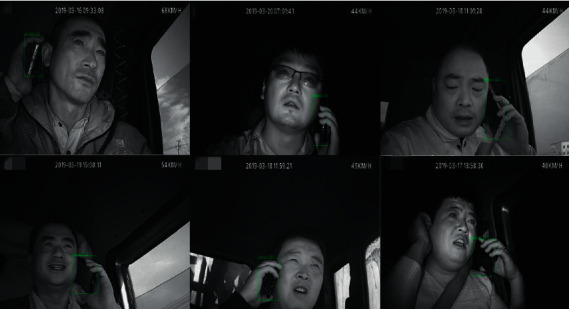
Detection results (threshold = 0.40).

**Figure 10:**
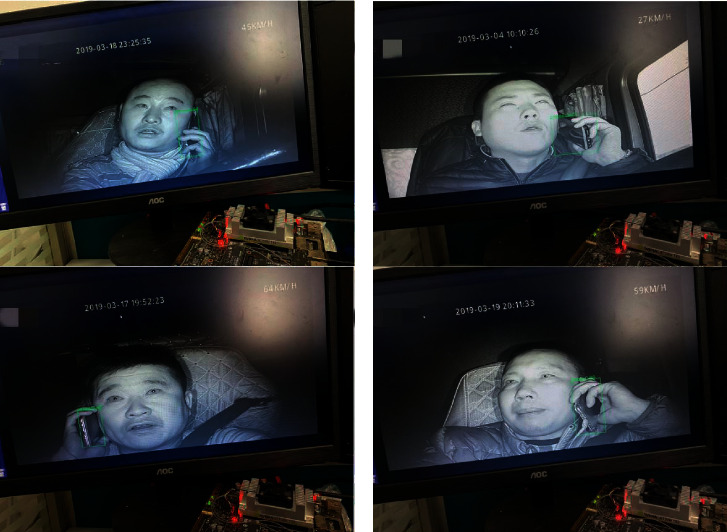
Illumination test.

**Figure 11:**
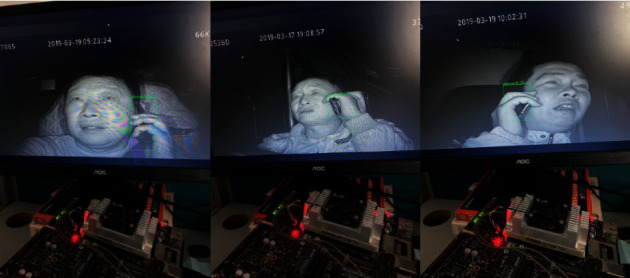
Occlusion test.

We conducted comparative experiments from the five aspects given below, in order to prove that the proposed model and algorithm have better detection effect, especially in the detection of small objects:

1. This section makes a comparison of the effects of different combination models by controlling variables and using different basic networks and SSD structures. The base network experiment was conducted on KITTI datasets using a combination of MobileNet, MobileNetV2 and MobileNet-I, and SSD structures.
2. The results are shown in Table 2. Comparing the results of lines 1 and 2, it is evident that when SSD is used in all detection networks and MobileNetV2 is used as the basic network, the network model size can be effectively reduced by 3.3 MB, and the detection accuracy is unaffected. Compared to lines 2 and 3, the results show MobileNet-I as the basic network, compared to MobileNetV2. By comparing the results of lines 2 and 3, it can be seen that MobileNet-I, as the basic network, has more 5x5 depth separable convolution with a slightly reduced model size compared with MobileNetV2, while imitating the inception structure to introduce SinConv convolution unit, and the model size is slightly reduced. This suggests that the depth of the network reaches a certain degree—a 5 × 5 convolution kernel than two 3 × 3 convolution kernels with less amount of calculation. In addition, MobileNet-I as a base network has a higher detection accuracy than MobileNetV2, with MAP improving by 1.3%, indicating that MobileNet-I is also better than MobileNetV2 in feature extraction.
3. In this section, experiments were carried out on KITTI, VOC, and Safe_Imgs datasets using several popular lightweight object detection networks—MobileNet-SSD, MobilenetV2-SSDLite, and LMS-DN—as proposed in this paper.
4. The experimental results on the VOC and KITTI data sets are shown in Tables 3 and 4. It can be seen from the tables that the basic network, MobilenetV2, can reduce the size of the model while improving the accuracy. By comparing the results of the first and second lines, it can be seen that MobilenetV2-SSdLite reduces the size of the model while attaining higher accuracy than MobileNet-SSD. By comparing the results of lines 2 and 3, it can be seen that when compared with MobilenetV2-SSDLite, MAP of LMS-DN proposed in this paper improved by 1.4% and 2.6%, respectively, under the condition that FPS of model size was slightly enlarged while actual FPS slightly decreased. Higher recognition accuracy was obtained with fewer parameters and calculation costs in comparison to MobilenetV2-SSDLite. The effect diagram of MobilenetV2-SSDLite and LMS-DN on KITTI dataset is shown in Figure 7. As can be seen from the diagram, under MobilenetV2-SSDLite, many cars with small proportions in the figure have not been detected, whereas when LMS-DN improved after SSDLite, there has been a great improvement on the detection of small target objects, and target vehicles at a long distance can be detected.
5. The experimental results on the Safe_Imgs data set are shown in Table 5. LMS-DN achieves 86.2% accuracy, 4.9% higher than MobileNet-SSD and 3.5% higher than MobileNetV2-SSDLite at the cost of less model size. At the same time, there are varying degrees of improvement in the accuracy rate and recall rate.
6. According to the above experimental results, improvements to SSDLite cause LMS-DN to detect smaller target objects than MobilenetV2-SSDLite.
7. To analyze the performance of MobileNetV2-SSDLite and LMS-DN models, the Safe_Imgs dataset was used to compare the accuracy of the two models under different thresholds under the same experimental conditions in this section.
8. As shown in Figure 8, it can be seen that the detection performance of MobileNetv2-SSDLite decreases significantly when the threshold increases gradually from 0.25 to 0.55. However, for LMS-DN, when the threshold value is 0.55, the accuracy rate still reaches 82.60%, indicating that LMS-DN has a good anti-interference capability.  Figure 9 shows the detection results of a threshold value of 0.4.
9. The previous part proves that LMS-DN performs better than other popular lightweight networks through experiments. In this section, there is a discussion on the network model that is more suitable for porting to the embedded development board, NVIDIA Jetson TX2.
10. This experiment compares the average detection time of LMS-DN and three other models for a single image on Jetson TX2, as shown in Table 6. Although the average detection time of LMS-DN is only 12 MS longer than that of MobileNetV2-SSDLite, it can still meet the requirements of real-time detection of mobile devices. Compared with the other two models, LMS-DN can achieve higher detection accuracy at almost the same speed. In summation, LMS-DN stands out among many models because of both high precision and speed, which are more suitable for the transplantation of embedded devices.
11. LMS-DN was tested on NVIDIA Jetson TX2 for different lighting and obstacle occlusion images in this section.
12. Different lighting tests were carried out on the NVIDIA Jetson TX2 development board. Some of the renderings are shown in Figure 10, where (a) is the low-light detection result diagram, and (b) is the high-light detection result diagram. It can be seen from the figure that the detection accuracy of LMS-DN is still very high under the conditions of strong and weak light.  Table 7 shows the statistical results of three different lighting types. In addition, obstacle-blocking test was carried out on the development board, and some picture effects were obtained, as shown in Figure 11, and the statistical results are shown in Table 8. LMS-DN can also accurately detect the mobile phone with the target object when the palm blocks most of it. To sum up, according to the statistical data, the LMS-DN model proposed in this paper can not only overcome the influence of strong and weak light, but it can also complete the real-time detection of targets with high accuracy in case of certain interference of obstacles.

## 6. Conclusions

This paper proposes a lightweight network LMS-DN for the driver's active safety prevention and control system, which can detect effectively and in real time on embedded devices. The network is improved on the basis of MobileNetV2-SSDLite and tested using KITTI, VOC, and Safe_Imgs datasets. The experimental results show that considering the factors such as accuracy, speed, and model size, the lightweight driver handheld call detection network proposed in this paper is more accurate and has stronger anti-interference in the detection of small target objects than the previous lightweight object detection networks. In future work, more real-time tests will be conducted on the embedded platform for different driving situations.

## Figures and Tables

**Table 1:** Structure of MobileNetV2.

Input	Operator	*t*	*c*	*n*	*s*
224^2^ × 3	Conv2d	—	32	1	2
112^2^ × 32	Bottleneck	1	16	1	1
112^2^ × 16	Bottleneck	6	24	2	2
56^2^ × 24	Bottleneck	6	32	3	2
28^2^ × 32	Bottleneck	6	64	4	2
14^2^ × 64	Bottleneck	6	96	3	1
14^2^ × 96	Bottleneck	6	160	3	2
7^2^ × 160	Bottleneck	6	320	1	1
7^2^ × 320	Conv2d 1 × 1	—	1280	1	1
7^2^ × 1280	avgpool 7 × 7	—	—	1	—
1 × 1 × 1280	Conv2d 1 × 1	—	*k*	—	—

**Table 2:** Detection results of different basic networks.

Network	Dataset	Model size (MB)	mAP (%)
MobileNet-SSD	KITTI	25.1	46.8
MobileNetV2-SSD	KITTI	21.8	47.0
MobileNet-I-SSD	KITTI	21.5	48.3

**Table 3:** Results on the VOC0712 dataset.

Network	Dataset	Model size (MB)	mAP (%)
MobileNet-SSD	VOC0712	23.3	72.3
MobileNetV2-SSDLite	VOC0712	19.7	72.6
LMS-DN	VOC0712	20.5	76.2

**Table 4:** Results on the KITTI dataset.

Network	Dataset	Model size (MB)	FPS	mAP (%)
MobileNet-SSD	KITTI	25.1	53	46.8
MobileNetV2-SSDLite	KITTI	21.6	59	47.1
LMS-DN	KITTI	22.5	58	49.7

**Table 5:** Results on the Safe_Imgs dataset.

Network	Dataset	Model size (MB)	FPS	Accuracy (%)	Precision (%)	Recall (%)
MobileNet-SSD	Safe_Imgs	18.6	59	81.3	90.6	80.6
MobileNetV2-SSDLite	Safe_Imgs	17.5	65	82.7	92.8	83.5
LMS-DN	Safe_Imgs	17.9	63	86.2	96.3	85.2

**Table 6:** Average time of single image detection by different models on Jetson TX2.

Network	Average time
VGG16-SSD	246
MobileNet-SSD	58
MobileNetV2-SSDLite	50
LMS-DN	56

**Table 7:** Results of different illumination detection.

Degree of light	Accuracy (%)	Number of test images
Normal	86.2	80
Bright	72.5	80
Weak	77.1	80

**Table 8:** Results of obstruction detection.

	Accuracy (%)	Number of test images
Normal	86.2	80
Partially occluded	70.8	80

## Data Availability

The data used to support the findings of this study are available from the corresponding author upon request.
